# Formation of Nano-Fibrous Patterns on Aluminum Substrates via Photolithographic Fabrication of Electrospun Photosensitive Polyimide Fibrous Membranes

**DOI:** 10.3390/nano12162745

**Published:** 2022-08-10

**Authors:** Yan-shuang Gao, Xi Ren, Xuan-zhe Du, Zhen-zhong Wang, Zhi-bin He, Shun-qi Yuan, Zhen Pan, Yan Zhang, Xin-xin Zhi, Jin-gang Liu

**Affiliations:** 1 Beijing Key Laboratory of Materials Utilization of Nonmetallic Minerals and Solid Wastes, National Laboratory of Mineral Materials, School of Materials Science and Technology, China University of Geosciences, Beijing 100083, China; 2 RAYITEK Hi-Tech Film Company, Co., Ltd., Shenzhen 518105, China

**Keywords:** photosensitive polyimide, electrospinning, nano-fibrous membrane, photolithography, pattern

## Abstract

The formation of polymeric micro-patterns on various substrates via a photolithography procedure has been widely used in semiconductor fabrication. Standard polymer patterns are usually fabricated via photosensitive polymer varnishes, in which large amounts of potentially harmful solvents with weight ratios over 50 wt% have to be removed. In the current work, a novel pattern-formation methodology via solvent-free electrospun photosensitive polymeric fibrous membranes (NFMs) instead of the conventional photosensitive solutions as the starting photoresists was proposed and practiced. For this purpose, a series of preimidized negative auto-photosensitive polyimide (PSPI) resins were first prepared via the two-step chemical imidization procedure from the copolymerization reactions of 3,3′,4,4′-benzophenonetetracarboxylic- dianhydride (BTDA) and two *ortho*-methyl-substituted aromatic diamines, including 3,3′,5,5′-tetramethyl-4,4′-diaminodiphenylmethane (TMMDA) and 3,7-diamino-2,8-dimethyl- dibenzothiophene sulfone (TSN). The derived homopolymer PI-1 (BTDA-TMMDA) and the copolymers, including SPI-2~SPI-6, with the molar ratio of 5~25% for TSN in the diamine units, showed good solubility in polar solvents. Then, a series of PSPI NFMs were fabricated via standard electrospinning procedure with the developed PSPI solutions in *N,N*-dimethylacetamide (DMAc) with a solid content of 25 wt% as the starting materials. The derived PSPI NFMs showed good thermal stability with 5% weight loss temperatures higher than 500 °C in nitrogen. Meanwhile, the derived PSPIs showed good photosensitivity to the ultraviolet (UV) emitting wavelengths of i-line (365 nm), g-line (405 nm) and h-line (436 nm) of the high-pressure mercury lamps in both forms of transparent films and opaque NFMs. Fine micro-patterns with a line width of around 100 μm were directly obtained from the representative SPI-4 NFM via standard photolithography procedure.

## 1. Introduction

Polymeric nano-fibrous membranes (NFMs) have been paid increasing attention in high-tech applications, such as energy, environment, microelectronics, optoelectronic display, and so on, due to the intrinsic high specific surface area, high porosity, high optical reflectivity, and low dielectric constants [1,2,3,4,5]. Electrospinning and electrospraying have been becoming one of the most efficient types of procedures for producing polymeric NFMs after many decades of basic and industrial research [6,7,8,9,10,11].

**Figure 1:**
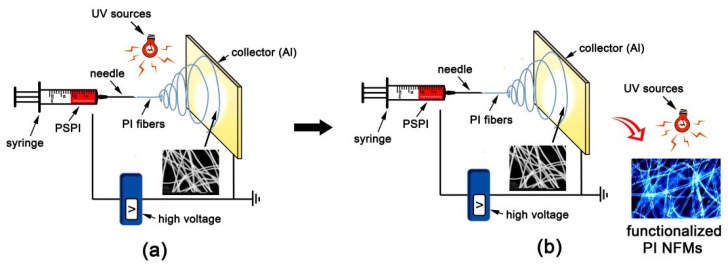
Ultraviolet-assisted electrospinning or electrospraying procedures for PI NFMs. (**a**) “UVAES”; (**b**) modified “UVAES”.

Recently, the post-modification of NFMs so as to achieve various functionalities has greatly expanded the wide applications of NFMs. Sagitha et al. reviewed the post-modification strategies of polymeric electrospun membranes in 2018 [12]. Among the various physical and chemical post-treatment methodologies, photochemical, especially ultraviolet (UV), treatments have been thought to be one of the most promising procedures for the functionalization of polymeric NFMs due to the abundant interactions between the polymer and the UV exposure. In addition, the UV modification procedure belongs to the “green” technique in the fabrication of polymeric NFMs due to its solvent-free nature [13]. Thus, the combination of the UV technique and the electrospinning or electrospraying fabrications of polymeric NFMs have made remarkable progress in the past decade. Jin et al. reported the electrospun photosensitive nano-fibers derived from poly (3-hexylthiophene) (P3HT) and polycaprolactone (PCL) [14]. The obtained photosensitive scaffold showed good stimulating sensitivity to the wavelength of white light-emitting diodes (LED) (421~670 nm). The good comprehensive properties made the P3HT/PCL NFMs good candidates in photocurrent therapy for skin regeneration. Wang and coworkers reported the highly sensitive photo-responsive polyamide 6 NFM membrane containing embedded spiropyran components [15]. The reversible isomerization nature of spiropyran-merocyanine units meant that the fabricated NFM patterns could be written, erased, and rewritten using UV light and visible light; thus, applications in counterfeit-proof areas might be found. Similarly, Khatri et al. reported the polyurethane (PU) and polystyrene (PS) NFMs containing the photosensitive spiropyran dye [16]. The resultant NFMs showed the photo-switching behaviors upon alternate irradiation with UV at the wavelength of 365 nm for coloration and with the visible light for de-coloration on a designed photomask. The developed polymers could potentially be used for data recording/erasing applications. In our previous work, a new procedure, the “ultraviolet-assisted electrospinning or electrospraying (UVAES)” technique, as shown in Figure 1a, was developed and adopted to fabricate the polyimide (PI) NFMs by using the organo-soluble and preimidized negative photosensitive polyimides (PSPIs) containing benzophenone units as the starting electrospinning solutions [17,18,19,20,21]. During the fabrication, photocrosslinking reactions induced by the photosensitive benzophenone units [22,23] in-situ occurred in the molecular structures of the ultrafine PI fibers with the UV exposure; thus, high-temperature and solvent-resistant polyimide (PI) NFMs were successfully obtained in one step. This technique uses organo-soluble PSPIs instead of traditional poly (amide acid) (PAA) precursors as the electrospinning solutions to fabricate the PI NFMs. Thus, it could efficiently avoid the yellowing, pinholes, adhesion, and other defects in the final PI NFMs caused by the high-temperature imidization and dehydration reactions of PAA at elevated temperatures up to 350 °C [24]. Although the UVAES technique could afford PI NFMs with fine micro- and macrostructures, the UV post-modification for the PI NFMs is difficult due to the highly crosslinking structures.

In the current work, a modified “UVAES” technique was developed, as shown in Figure 1b. It is different from the previously reported “UVAES” procedure (Figure 1a) in that the UV modification was post-performed instead of the in-situ execution. The same part is that both of the procedures use organo-soluble and preimidized negative PSPIs as the starting electrospinning solutions. The UV post-modification, specifically the patterning processing via the photolithography of the electrospraying photosensitive PI NFMs, was carried out in this work. The effects of the molecular structures of the starting PSPIs, the electrospraying conditions, and the photolithographic conditions on the micro-morphologies of the PI NFM patterns were investigated in detail.

## 2. Materials and Methods

### 2.1. Materials

Highly pure 3,3′,4,4′-benzophenonetetracarboxylic dianhydride (BTDA, UP grade) was purchased from Evonik Degussa Corp. (Frankfurt, Germany) and dried in vacuum at 180 °C overnight prior to use. 3,3′,5,5′-Tetramethyl-4,4′-diaminodiphenylmethane (TMMDA) was purchased from Tokyo Chemical Industry (TCI) Co., Ltd. (Tokyo, Japan) and used as received. 3,7-Diamino-2,8-dimethyldibenzothiophene sulfone (TSN) was purchased from Wakayama Seika Holdings Co., Ltd. (Wakayama, Japan) and used directly. Ultra-dry *N*-methyl-2-pyrrolidone (NMP), *N,N*-dimethylformamide (DMF), and *N,N*-dimethylacetamide (DMAc) with water contents lower than 50 ppm were obtained from Beijing Innochem Sci&Technol Co. Ltd. (Beijing, China) and used directly. The other solvents, including γ-butyrolactone (GBL) and cyclopentanone (CPA) were obtained from Sinopharm Chemical Reagent Co. Ltd. (Shanghai, China) and purified by distillation prior to use.

### 2.2. Measurements

Inherent viscosities of the PI resins were measured using an Ubbelohde viscometer (Pingxuan Scientific Instrument Co. Ltd., Shanghai, China) with a 0.5 g dL ^−1^ NMP solution at 25 °C. Absolute viscosity of the PI solutions was measured using a DV-II+ Pro viscometer (Brookfield, Ametek, MA, USA) at 25 °C. The number average molecular weight (*M_n_*) and weight average molecular weight (*M_w_*) of the PI resins were measured using a gel permeation chromatography (GPC) system (Shimadzu, Kyoto, Japan). The solubility of the PI resins was investigated by mixing 1.0 g of the PI resins and 9.0 g of the solvent tested (10 wt% solid content) and then stirred for 24 h at room temperature. The solubility was determined visually as three grades: completely soluble (++), partially soluble (+), and insoluble (−), wherein complete soluble indicates a homogenous and clean state without phase separation, precipitation or gel formation, and insoluble indicates no change in the appearance of the resin. Attenuated total reflectance Fourier transform infrared (ATR-FTIR) spectra of the PI NFMs were recorded on a Iraffinity-1S FT-IR spectrometer (Shimadzu, Kyoto, Japan). Thermogravimetric analysis (TGA) and the derivative TGA (DTG) of the PI NFMs were performed on a Q50 thermal analysis system (New Castle, Delaware, USA) at a heating rate of 10 °C min^−1^ in nitrogen. Thermo-mechanical analysis (TMA) of the PIs was recorded on a TMA402F3 thermal analysis system (NETZSCH, Selb, Germany) by using films instead of fibrous membranes. The PI films with a thickness of 25 ± 0.5 μm were prepared according to our previous work [25]. The thermal scanning mode ranges from 50 to 450 °C at a heating rate of 5 °C min^−1^ in nitrogen atmosphere. The coefficients of linear thermal expansion (CTE) values of composite films were recorded in the range of 50~250 °C. Ultraviolet-visible (UV-Vis) spectra of the PI NFMs were recorded on a Hitachi U-3210 spectrophotometer (Tokyo, Japan). Wide-angle X-ray diffraction (XRD) of the PI NFMs was conducted on a Rigaku D/max-2500 X-ray diffractometer (Tokyo, Japan). The micro-morphologies of the PI patterns were detected via a JSM-6700F (JEOL, Tokyo, Japan) field emission scanning electron microscopy (FE-SEM). The CIE (Comission Internationale del’ Eclairage, Vienna, Austria) Lab optical parameters of the PI NFMs were measured using an X-rite color i7 spectrophotometer (Michigan, USA). For the color parameters, L^*^ is the lightness, where 100 means white and 0 implies black. A positive a^*^ means a red color, and a negative one indicates a green color. A positive b^*^ means a yellow color, and a negative one indicates a blue color. The whiteness values of the PI NFMs were calculated as Equation (1), where WI standards for whiteness index, L^*^ standards for the index of lightness, a^*^ and b^*^ stand for chromaticity coefficient.

WI = 100 − [(100 − L^*^)^2^ + a^*2^ + b^*2^]^1/2^ (1)

Effects of the structural modification on the photocrosslinking behaviors of the PIs were evaluated as follows. The tested PI solution with a solid content of 30 wt% was spin-coated onto potassium bromide (KBr) sheets (1.0 cm × 1.0 cm × 2 mm) at 700 rpm for 30 s using a spin-coater (MS-B100, Mikasa Co. Ltd., Hiroshima, Japan). Then, the KBr sheets were prebaked at 80 °C for 0.5 h on a digital hotplate (NA-1A, As-One Co., Ltd., Osaka, Japan). The thickness of the obtained PI coatings was 5 ± 0.5 μm The KBr sheets were exposed with a UV light source (IMS-811A-0606, IUVOT Co., Ltd., Jiangsu, Changzhou, China, power density: 400 mW/cm^2^). The distance between the UV source and the PI samples was 1.0 ± 0.1 cm. The UV exposure time was set to be 0.5, 1.0, 2.0 and 3.0 min. After exposure, the ATR-FTIR spectra of the PI samples were detected.

The photolithographic patterning procedures of the PI NFMs were investigated as follows. The PI NFM with a thickness of 20.0 ± 1.0 μm on the aluminum substrate was covered with a photomask containing the “CUGB” pattern together with the corresponding numbers (line width with the unit of a micrometer). Then, the PI NMF/mask system was exposed for 60 s with a high-pressure mercury lamp equipped with a UV-LED system (Model: IMS-811A-0606, IUVOT Co., Ltd., Jiangsu, Changzhou, China) with the main ultraviolet wavelength of i-line (365 nm), g-line (405 nm), and h-line (436 nm). Then, the exposed PI NFM was developed in DMF for 5 s, after which the sample was immersed in butyl acetate for 10 s. At last, the PI NFM was baked at 120 °C for 60 min. The photolithography results were observed by SEM measurement. The thickness of the pattern was measured with SEM measurement or Dektak XTL Stylus Profilometer (Tucson, AZ, USA).

### 2.3. Synthesis of Photosensitive PI Resins

One PI homopolymer, PI-1 and five PI copolymers were synthesized according to the formulations shown in Table 1. The detailed synthesis procedure could be illustrated by the synthesis of SPI-4, in which the molar ratio of TMMDA to TSN was 85:15 in the diamine moiety. TMMDA (10.8107 g, 0.0425 mol) and TSN (2.0576 g, 0.0075 mol) together with ultra-dry DMAc (80.0 g) were added to a 250 mL three-necked flask equipped with a mechanical stirrer, a nitrogen inlet, and a cold bath. After stirring at 10 °C for 0.5 h, a clear diamine solution was obtained. Then, BTDA (16.1115 g, 0.05 mol) and DMAc (6.9 g) were added into the obtained diamine solution to afford a polymerization system with a solid content of 25 wt%. After stirring at 10 °C for 4 h, the cold bath was removed, and room temperature was maintained for another 20 h for the reaction system so as to increase the molecular weights of the obtained poly (amic acid) (PAA) precursor. Subsequently, the dehydrating agent of acetic anhydride (25.5 g, 0.25 mol) together with the catalyst of pyridine (15.8 g, 0.20 mol) was added to the PAA solutions. The polymerization system was stirred at room temperature for another 24 h to finish the transition from the PAA precursor to the final SPI-4 via the dehydrating imidization. The obtained viscous SPI-4 solution was slowly poured into the aqueous ethanol (70 vol%) to afford the pale-yellow silky resin. The resin was filtered and dried at 80 °C in vacuum for 24 h (Yield: ~96%). FTIR (cm^−1^): 2924, 2866, 1778, 1716, 1635, 1373, 1157, and 729.

The other PI resins were similarly prepared, except that the molar ratio of the TMMDA/TSN was set to be 100:0 for PI-1, 95:5 for SPI-2, 90:10 for SPI-3, 80:20 for SPI-5, and 75:25 for SPI-6, respectively.

PI-1. FTIR (cm^−1^): 2924, 1778, 1716, 1670, 1601, 1362, 1211, 1092, 849, and 721.

SPI-2. FTIR (cm^−1^): 2924, 1778, 1724, 1674, 1636, 1485, 1369, 1157, and 729.

SPI-3. FTIR (cm^−1^): 2924, 2866, 1778, 1717, 1636, 1373, 1157, and 729.

SPI-5. FTIR (cm^−1^): 2924, 2866, 1778, 1732, 1674, 1632, 1373, 1157, and 729.

SPI-6. FTIR (cm^−1^): 2924, 2866, 1778, 1728, 1631, 1373, 1157, and 729.

### 2.4. Fabrication of PI NFMs via Electrospinning

The PI and SPI NFMs were fabricated via the standard electrospinning procedure by using the PI solutions as the starting materials. For comparison, the same solid content of 30 wt% was used in the current work so as to maintain the similar thickness of the derived NFMs as accurately as possible. The electrospinning parameters, including the voltage (15 kV), the inner diameters of the spinnerets (0.21 mm), the distance between the spinneret to the alumina foil collector (15 cm), the speed of the rotating drum collector (1000 rpm), and the temperature (20 ± 2 °C) and relative humidity (30 ± 5 %), were also controlled to be the same. The detailed electrospinning fabrication could be illustrated by the preparation of SPI-4 NFM. The dried SPI-4 resin obtained in Section 2.3 was dissolved in DMAc at room temperature with the solid content of 30 wt%. A yellow solution with the absolute viscosity of 3377 mPa s was then obtained. The SPI-4 solution was filled into a 5 mL syringe and squeezed out through a spinneret by a syringe pump at a speed of 0.2 mL/h. The voltage was applied between the syringe and the rolling drum collector. Ultrathin SPI-4 fibers or beads deposited on the aluminum foil substrates. After the electrospinning, the free-standing SPI-4 NFM was obtained by being peeled from the aluminum substrate and then dried in an air-circulated oven at 120 °C for 1h to remove the absorbed moisture and the residual solvent.

The other NFMs were prepared according to a similar procedure. The free-standing PI NFMs were used to test the FTIR, thermal properties, optical reflectance, and CIE Lab parameters. The PI NFMs on the aluminum substrates were used to evaluate the photolithography patterning properties.

## 3. Results and Discussion

### 3.1. PI Resin Synthesis and Electrospun PI NFMs Preparation

The standard PI-1 (BTDA-TMMDA) represents a type of auto-sensitive negative PSPI developed in the 1980s, which is intrinsically sensitive to the emitting wavelength of a high-pressure mercury lamp (365~436 nm) without any additional photoactive compounds [26]. In such PSPI, the benzophenone units in the BTDA moiety will be excited by the UV exposure to form the triplet, which will capture the hydrogen atoms in the methyl substituents *ortho* to the amino groups in the TMMDA moiety. This will lead to the intra- and intermolecular photocrosslinking reactions, making the UV-exposed parts insoluble in the developer (DMF or CPA) [27]. Thus, negative patterns to the photomask will be obtained. In view of the several intrinsic defects of the pristine PI (BTDA-TMMDA) system, such as the relatively low photo-efficiency, low optical transmittance to the UV light sources, the high coefficient of linear thermal expansion (CTE), and so on, various modifications via incorporation of the third or more monomers have been reported in the past decades [28,29]. Most of the above modification works are to improve the comprehensive properties of the pristine PI (BTDA-TMMDA) system with the preconditions of maintaining the inherent photosensitivity. In the current work, a third monomer, TSN diamine, was used to copolymerize with TMMDA to afford the modified PSPI with improved properties. On the one hand, the TSN diamine contains two *ortho*-substituted methyl groups to the amino groups in one molecular unit. Thus, the photosensitivity of the derived co-polyimides might be maintained to a great extent because the photoreaction efficiency of such types of PSPIs was positively correlated with the numbers of alkyl substituents in the repeating units of the polymers. Secondly, the TSN diamine has sulfone groups in the structure, whose bulky and electron-withdrawing nature might endow the derived PIs with enhanced solubility in organic solvents and improved optical transparency. At last, the TSN diamine has a ring-fused structure, which might provide high thermal stability to the derived PIs. These are all desirable properties for the practical applications of the PIs.

**Figure 2:**
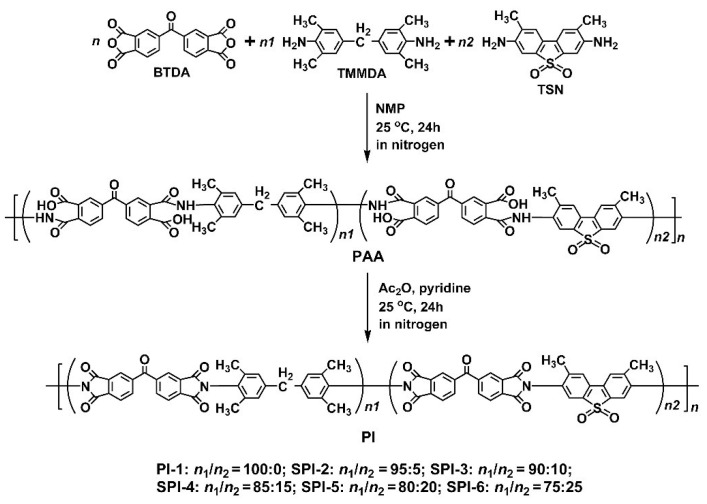
Synthesis of PI-1 (BTDA-TMMDA) and SPI copolymer resins.

The detailed synthesis procedure for the PIs is shown in Figure 2. All the reaction systems remained homogeneous, and no gelling or precipitating phenomena were observed, indicating the good solubility of the derived PI resins in the reaction medium. Flexible and tough silky PI resins were nearly quantitatively obtained after precipitating the PI solutions into the poor solvent of aqueous ethanol.

First, the inherent viscosities ([*η*]_inh_) and molecular weights of the PI and SPI resins were detected, and the results are shown in Table 2. The copolymer PI (SPI-2~SPI-6) resins showed the (*ƞ*)_inh_ values in the range of 0.47~0.78 dL g^−1^, the number of average molecular weights (*M_n_*) in the range of (2.87~4.70) × 10^4^ g mol^−1^, and the weight average molecular weights (*M_w_*) in the range of (5.13~8.83) × 10^4^ g mol^−1^, respectively. These values are all correspondingly lower than those of the pristine PI-1 (BTDA-TMMDA) system ((*ƞ*)_inh_ = 1.00 dL g^−1^, *M_n_* = 5.06 × 10^4^ g mol^−1^), indicating that incorporation of the TSN monomer decreased the molecular weights of the polymers. This might be due to the decreased reactivity of the TSN diamine due to the existence of the electron-withdrawing sulfone groups. Nevertheless, the current co-polyimides possessed acceptable molecular weights for the following NFM fabrications.

**Figure 3:**
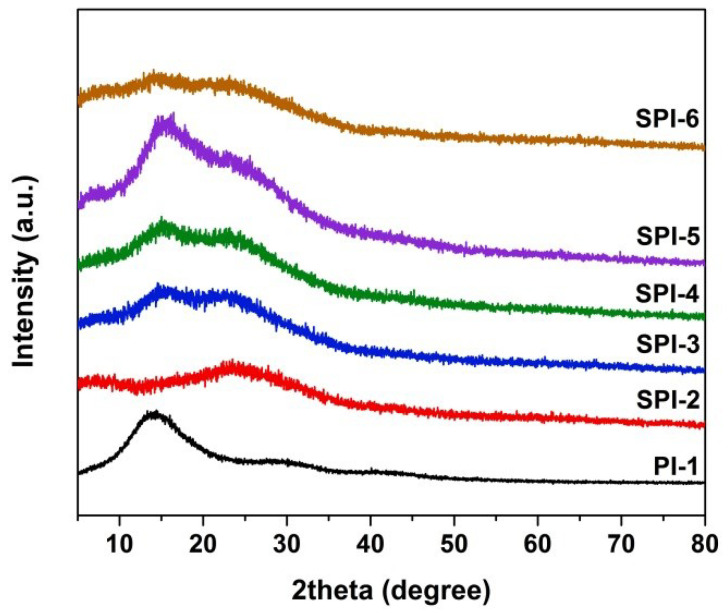
XRD spectra of PI-1 and SPI resins.

The solubility of the PI resins in some representative solvents was evaluated, and the results are presented in Table 2. As expected, all the PI resins were soluble in the polar aprotic solvents (NMP and DMAc) and ketone-type solvent (CPA) at room temperature at a solid content of 10 wt%. All of them were resistant to the ester-type solvent, such as GBL. Undoubtedly, the synergistic effects of the multi-substituted methyl groups and the bulky sulfone units efficiently prevented the ordered packing of the molecular chains in the polymers. This structural feature endowed the PI resins amorphous nature, as could be proven by the XRD spectra of the polymers shown in Figure 3. When the molar ratio of the TSN in the diamine moiety was lower than 10% (SPI-2 and SPI-3), they were also soluble in THF. Further higher loading of TSN resulted in the deterioration of the solubility in THF. Basically, the incorporation of the TSN component slightly decreased the solubility of the PI resins in organic solvents, which might be due to the highly conjugated and fused rings in the diamine. The good solubility of the PI resins in the tested solvents makes it possible to fabricate the PI NFMs using the PI solutions as the starting materials.

**Figure 4:**
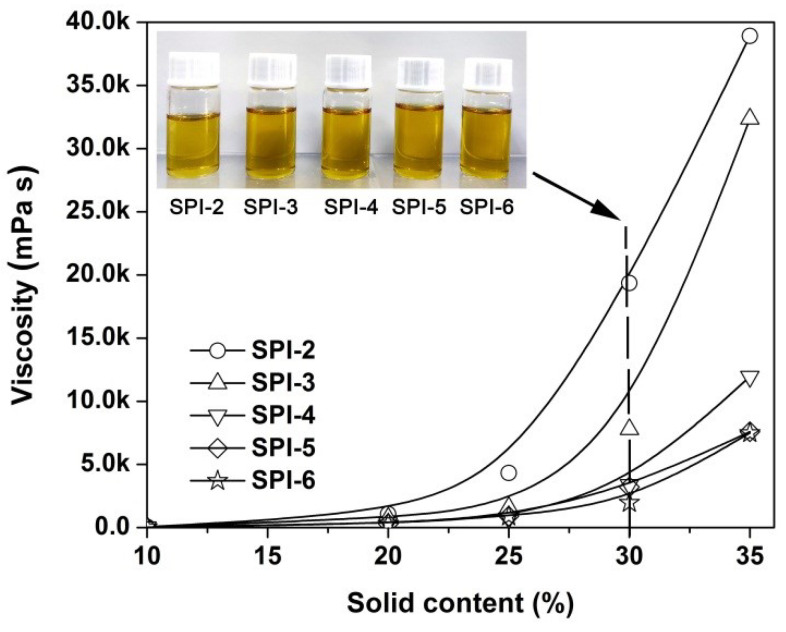
Relationship between the solid contents and the absolute viscosities of the SPI solutions. (Insert: appearance of the SPI solutions).

Although all the co-polyimide resins were soluble in the polar solvents, such as in DMAc, the dissolution behaviors might be quite different. Therefore, the correlations between the solid contents and the absolute viscosities of the PI solutions were quantitatively evaluated, as shown in Figure 4. The absolute viscosities of the PI solutions were determined by both the solubility of resins in the solvent and the molecular weights of the resins. Thus, the plot could reveal the dissolution behaviors of the PI resins. For example, at the same solid content of 30 wt%, the viscosities of the PI solutions increased with the order of SPI-6 < SPI-5 < SPI-4 < SPI-3 < SPI-2. This is in good agreement with the changing trend of *M*_n_ values for the PI resins. On the other hand, in order to achieve the same absolute viscosity for the PI solutions, much more resins with relatively low molecular weight have to be added. The information was beneficial for the selection of appropriate solution parameters for the electrospinning fabrication of the PI NFMs.

**Figure 5:**
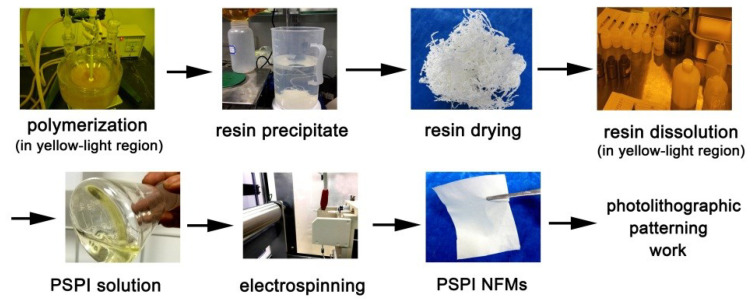
Electrospinning fabrication for the photosensitive PI and SPI NFMs.

Based on the evaluation results of the solution properties of the developed PI resins, a series of photosensitive PI NFMs were fabricated by a standard electrospinning procedure, as shown in Figure 5. In the current work, PI solutions with the same solid content of 30 wt% were used as the electrospinning solutions so as to achieve a similar thickness to the final NMFs. According to the data shown in Figure 4, the absolute viscosities of the PI solutions were 19,350 mPa s for SPI-2, 7773 mPa s for SPI-3, 3377 mPa s for SPI-4, 3199 mPa s for SPI-5, and 1957 mPa s for SPI-6, respectively. It can be anticipated that the different viscosities of the starting materials will affect the final micro-morphologies of the derived PI NFMs.

**Figure 6:**
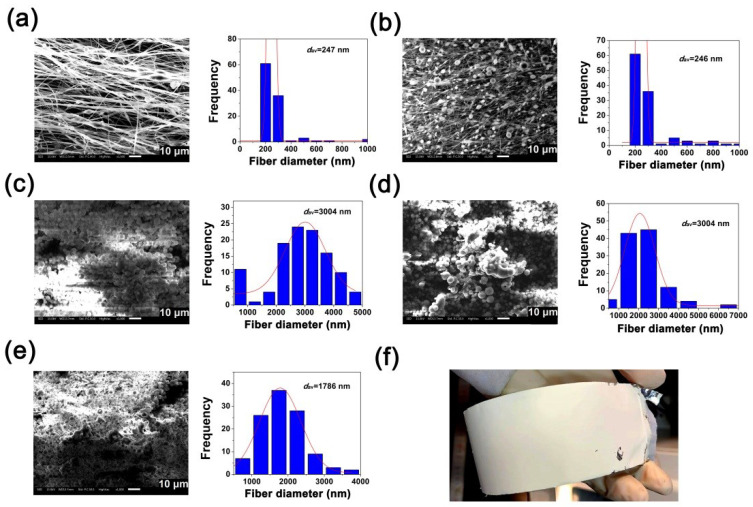
Microscopic (**a**–**e**) and macroscopic (**f**) morphologies of PI NFMs together with the average diameters of the fibers. (**a**–**e**) SEM images of SPI-2, SPI-3, SPI-4, and SPI-5 membranes, respectively; (**f**) Appearance of SPI-4 NFM.

Figure 6 shows the microscopic and representative macroscopic morphologies of the PI NFMs together with the average diameters of the fibers. It can be clearly seen from Figure 6a–e that as the viscosity (molecular weight) of the starting electrospinning solution decreased, the micro-morphologies of the PI NFMs gradually evolved from the filaments (SPI-2) to the combination of filaments and beads (SPI-3), and finally formed NFMs almost completely composed of the bead structures (SPI-4, SPI-5, and SPI-6). It has been well established in the literature that the micro-morphologies of the electrospun NFMs could be tailored by adjusting the solution parameters of the starting solutions [30]. Bead-like structures could often be achieved under the comprehensive actions of gravity force, electrical field force, and surface tension of the polymer droplets when the dilute solution was used as the electrospinning solution. The current phenomenon is in good agreement with the literature reports. The SPI-2 and SPI-3 NFMs mainly composed of fibers showed statistical average diameters (*d*_av_) of around 240 nm, whereas the SPI-4~SPI-6 NFMs, mainly composed of beads, showed much higher *d*_av_ values at the level of micrometers. The representative SPI-4 NFM showed a compact and pale-brown appearance, as shown in Figure 6f.

**Figure 7:**
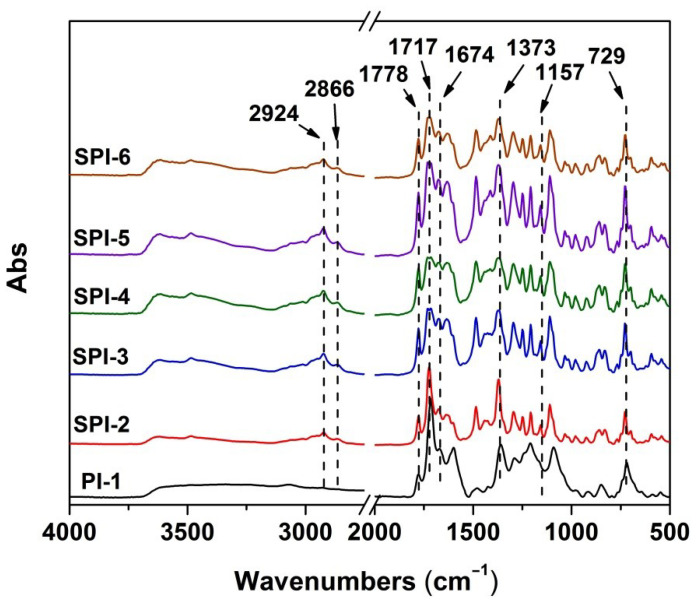
ATR-FTIR spectra of PI-1 and the SPI NFMs.

The chemical structures of the PI NMFs were confirmed by the AFR-FTIR spectra shown in Figure 7. First, all the characteristic absorptions of imide rings, including the asymmetrical and symmetrical carbonyl stretching vibrations at the wavenumber of 1778 and 1717 cm^−1^, respectively, the C-N stretching vibration at 1373 cm^−1^, and the in-plane bending vibrations of carbonyl peaks at 729 cm^−1^ were clearly detected in the spectra. Meanwhile, the characteristic absorptions of saturated C-H bonds in methyl groups at 2924 and 2866 cm^−1^ were also observed in all of the spectra. However, the characteristic symmetrical stretching vibrations of –SO_2_– at 1157 cm^−1^ were only detected in the spectra of SPI-2~SPI-6. It demonstrates the successful preparation of the target PIs.

### 3.2. Thermal Properties

**Figure 8:**
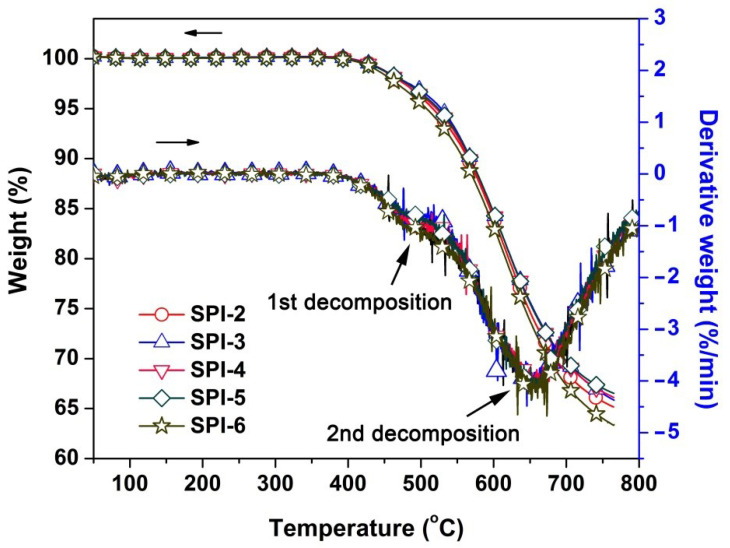
TGA and DTG curves of SPI NFMs.

The thermal stability of the PI NFMs was then evaluated by TGA measurements, and the results are listed in Table 3. It can be clearly detected from the TGA and derivative TGA (DTG) plots of the SPI NFMs shown in Figure 8 that all the polymers showed good stability before 400 °C, after which all the SPI NFMs gradually decomposed and revealed the 5% weight loss temperatures (*T*_5%_) in the range of 507.6~527.8 °C, the 10% weight loss temperatures (*T*_10%_) in the range of 558.3~568.4 °C, and the residual weight ratios at 750 °C (*R*_w750_) of 64.0~67.0%. All the thermal data of the SPI NFMs were a bit lower than those of the pristine PI-1 NFM when comparing the corresponding thermal data shown in Table 3. This is mainly due to the relatively low thermal stability of sulfone groups at elevated temperatures [31]. The SPI-6 NFM with the highest sulfone contents in the molecular structure showed the lowest thermal data among the polymers. In addition, according to the DTG plots, all the SPI NFMs showed two-stage thermal decomposition behaviors during the test. The first stage occurred around 450 °C, which might be due to the decomposition of methyl side chains or sulfone units, and the second stage was recorded around 650 °C, which could be ascribed to the decomposition of the main chains of the PIs.

**Figure 9:**
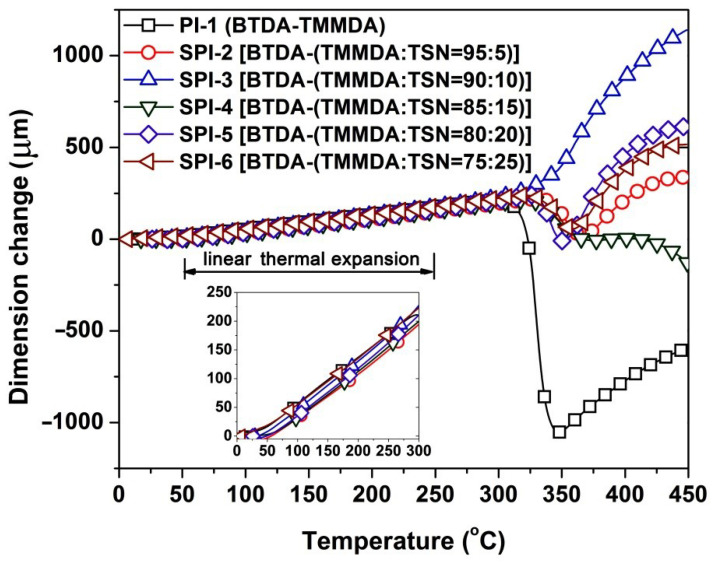
TMA curves of PI and SPI films. (Insert: locally enlarged TMA curves for clear demonstration with the same X- and Y-axis captions).

In addition to the TGA measurements for the PI NFMs, the structurally analogous PI films were also prepared for the TMA measurements in order to reveal the glass transition temperatures (*T*_g_) and linear coefficients of thermal expansion (CTE) of the polymers. Flexible and tough SPI films with good optical transparency and pale-yellow colors were obtained by the standard casting procedure from SPI solutions. Effects of the incorporation of TSN units on the glass transition behaviors and the high-temperature dimensional stability of the SPIs were studied. As could be seen from the TMA plots of the PI films shown in Figure 9, all the PI films showed thermally shrinking behaviors at elevated temperatures. For the high-temperature-resistant polymers containing rigid-rod units, this unusual shrinkage beyond the *T*_g_ is usually considered to be an indication of the orderly arrangement of the molecular chain segments in the polymers. After the ordered arrangements, the polymers will show the usual dimensional expansion with increasing temperatures. The turning points of this dimensional change are usually recorded as the *T*_g_ values of the polymers. According to the records, the PI films showed the *T*_g_ values higher than 330 °C. It meant that the incorporation of the TSN components did not deteriorate the high-temperature stability of the pristine PI-1. According to the CTE values of the polymers, incorporation of the TSN units slightly improved the dimensional stability of the PI-1 film at elevated temperatures. The CTE values of the polymers gradually decreased with the increasing contents of TSN in the polymers. The SPI-6 film showed the lowest CTE value of 50.9 × 10^−6^/K in the temperature range of 50~250 °C, which is lower than that of PI-1 (CTE = 56.5 × 10^−6^/K). This might be due to the ring-fused dibenzothiophene sulfone structure of TSN. The highly conjugated and fused nature of the TSN units efficiently prohibited the free motion of the molecular chain segments at high temperatures. In summary, the high-*T*_g_ and low-CTE features of the SPIs are all beneficial for their applications in advanced optoelectronic fabrications.

### 3.3. Optical Properties

**Figure 10:**
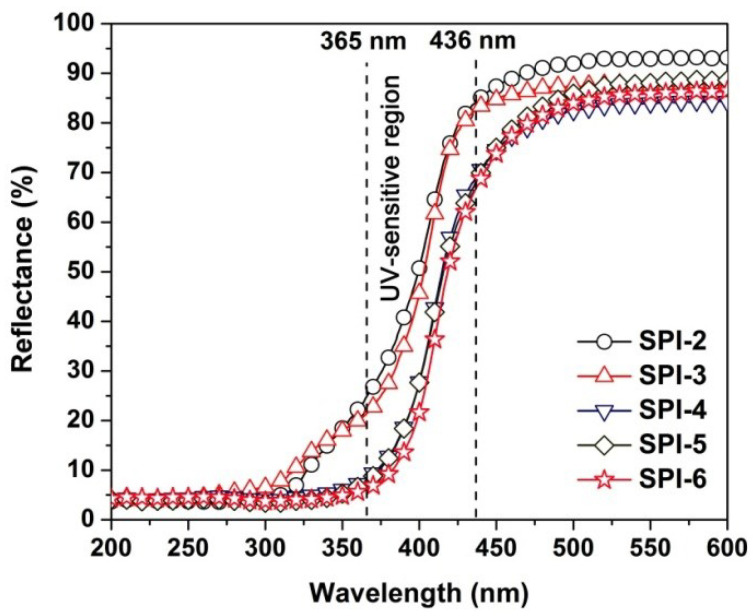
UV-Vis reflectance plots of PI membranes in the wavelength range of 200~600 nm.

The optical properties of the PI NFMs, including the optical reflectance, CIE Lab color parameters, photocrosslinking behaviors upon UV exposure, and photoimageable abilities via photolithography were systemically evaluated, and the related data are summarized in Table 4. First, the UV-Vis reflectance spectra of the SPI NFMs are shown in Figure 10. The SPI NFMs showed the optical reflectance values at the wavelength of 365 nm (*R*_365_) and 436 nm (*R*_436_) of 6.1~24.4% and 66.4~84.0%, respectively. These values were all correspondingly lower than those of the pristine PI-1 (*R*_365_ = 34.2%; *R*_436_ = 85.3%). This meant that more UV lights were absorbed by the SPI NFMs than that of PI-1. In addition, the optical reflectances of the PI NFMs decreased with the increasing TSN contents in the polymers. For example, the *R*_365_ values of the PI NFMs decreased with the order of PI-1 (34.2%) > SPI-2 (24.4%) > SPI-3 (21.1%) > SPI-4 (8.2%) > SPI-5 (7.6%) > SPI-6 (6.1%) The enhancement of the UV absorption might be due to the highly conjugated dibenzothiophene structure in the TSN units although the electron-withdrawing sulfone group was beneficial for reducing the UV absorption via prohibiting the charge transfer (CT) interactions in the molecular structures of the PIs. This enhancement of UV absorption might be advantageous for the photocrosslinking reactions in the SPI NFMs.

The increased UV absorbing ability via the incorporation of the TSN components also affected the CIE Lab color parameters of the PI NFMs, as shown in Table 4. Basically, with the increasing TSN contents in the polymers, the lightness (L^*^) of the NFMs gradually decreased while the yellow indices (b^*^) increased. This directly led to the decrease in the whiteness indices (WI) of the PI NFMs.

**Figure 11:**
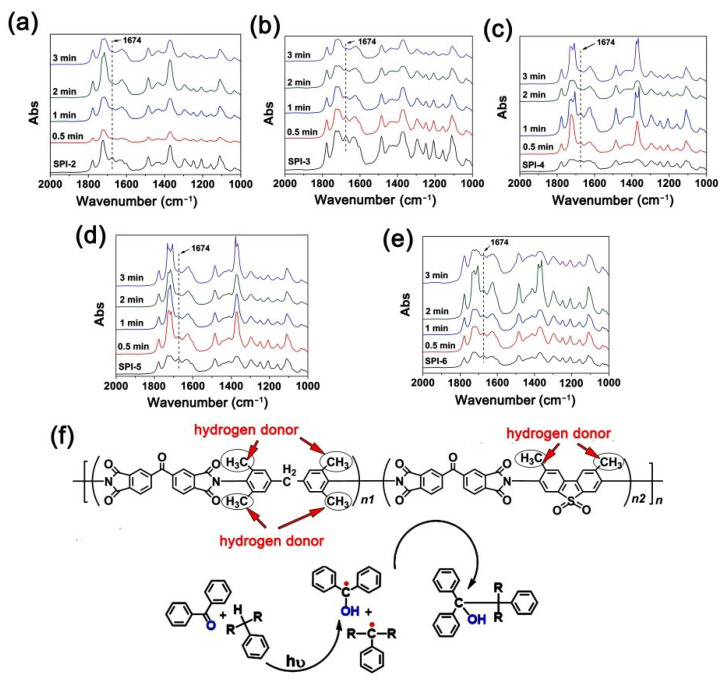
Photocrosslinking of benzophenone carbonyl at 1674 cm^−1^ with the exposure time (high-pressure mercury lamp, power: 400 mW/cm^2^). (**a**) SPI-2; (**b**) SPI-3; (**c**) SPI-4; (**d**) SPI-5; (**e**) SPI-6; (**f**) photocrosslinking mechanisms.

Secondly, the photocrosslinking behaviors of the SPIs upon UV exposure at different times (0~3 min) were investigated, and the results are shown in Figure 11. The investigation used the characteristic absorption strength of benzophenone carbonyl groups at the wavenumber of 1674 cm^−1^ in FTIR spectra as the indicator justifying the degree of photocrosslinking reactions. This is mainly due to the fact that once the carbonyl groups are excited by the UV light, the structure will change from >C=O to >Ċ-OH (triplet). Thus, the absorption strength at 1674 cm^−1^ in the FTIR spectra will decrease. If the absorption strength was 0, the photocrosslinking reaction was thought to be finished. The UV exposure times were set to be 0.5, 1, 2, and 3 min, respectively. It can be clearly observed from Figure 11a–e that all the copolymers could undergo the UV excitation reaction upon exposure, and all the photoreactions finished within 3 min according to the photocrosslinking mechanism shown in Figure 11f. It demonstrates that the SPI coatings showed good sensitivity to the emitting wavelengths (365~436 nm) of the high-pressure mercury lamp sources. This is quite beneficial for the following photolithography processing of the SPI NFMs.

**Figure 12:**
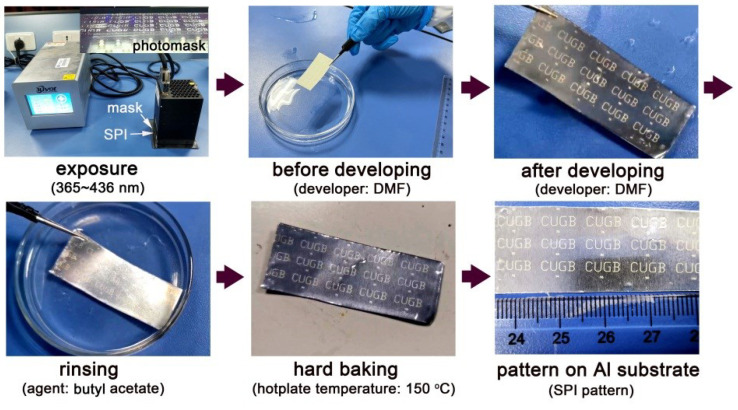
Photolithographic patterning process of SPI-4 NFM. (Insert: photomask).

At last, the photoimageable ability of the representative SPI-4 NFM was evaluated according to the process shown in Figure 12. First, the SPI-4 NFM with a thickness of around 20 μm on the aluminum substrate together with the photomask (“CUGB” patterns with different line widths) was exposed with an unfiltered high-pressure mercury lamp for 60 s. Then, the exposed PI NFM was developed in DMF for 5 s, after which the sample was rinsed in butyl acetate for 10 s. At last, the PI NFM was baked at 120 °C for 60 min.

**Figure 13:**
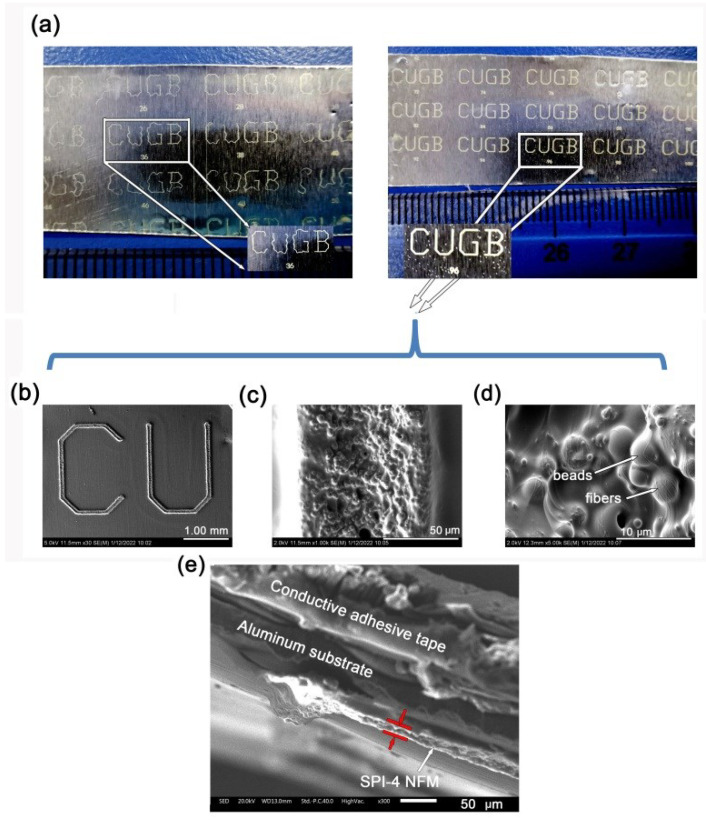
Macroscopic (**a**) and microscopic (**b**–**e**) morphologies of patterns formed by the photolithography process of SPI-4 NFM on aluminum substrate. (**a**) Left: “CUGB” patterns with the narrow line width; right: “CUGB” patterns with the wide line width (insert: “CUGB” pattern with the line width of 96 μm); (**b**–**d**): planar SEM photos of the locally enlarged patterns with the magnification times of 30 to 5000; (**e**): cross-sectional SEM photos of the SPI-4 pattern.

The thickness and micro-morphologies of the obtained SPI-4 NFM were checked, and the results are shown in Figure 13. According to the macro-morphologies of the NFM shown in Figure 13a, clear NFM patterns were first obtained on the aluminum substrates after the UV exposure and developing process. The unexposed region in SPI-4 NFM was totally dissolved into the DMF developer. It demonstrates that the methodology of forming patterns directly with the solvent-free PI NFM via the standard photolithography procedure is reasonable and feasible. The afforded PI NFM patterns showed the distorted profiles at the narrow line width (36 μm, left, Figure 13a) while fine profiles at the wide line width (96 μm, right, Figure 13a). It meant that although the photo-crosslinked SPI-4 NFM showed good resistance to the DMF developer, the patterns at thin line width showed slightly poor adhesion to the substrate. This resulted in the squiggly lines for the pattern. In the extreme case, the independent letter “B” floating in the DMF developer was observed in our experiments. This is because the ring nature of the letter “B” gives it a good initial shape in the developer. While the other letters, such as “C”, “U”, and “G”, become curved lines in the developer. When the line width became large, patterns with fine profiles were obtained. The thickness of the obtained SPI-4 NFM was about 15 ± 1 μm, according to the SEM measurements shown in Figure 13e. Given that the initial thickness for SPI-4 NFM before photolithography is about 20 μm, this means that the material maintains about 75% of the initial thickness during the photolithography process. Such a high thickness retention rate is mainly due to the intrinsic photosensitivity of the SPI-4. With no additional material, such as a large amount of photosensitizers, the photolithographic NFM showed a high ratio of thickness retention. This is very advantageous for the preparation of thick NFM-type patterns.

The micro-morphologies of the SPI-4 NFM patterns were further detected by SEM measurements, and the results are illustrated in Figure 13b–e. The patterns showed the a width of around 100 μm according to Figure 13c,d, which was a bit higher than that of the labeled value on the photomask (96 μm), which meant that the lines slightly swelled during the developing process. This could also be proven by the locally enlarged patterns with magnification times of 5000 (Figure 13d), in which the fusion of fibers and beads was clearly observed. In addition, the patterns basically maintained the porous feature of polymer NFM characterized by the high specific surface areas and good porosity.

The glass transition behaviors of the SPI-4 pattern were detected by the differential scanning calorimetry (DSC) (TA-Q 100, New Castle, DE, USA) measurements and no obvious *T*_g_ was detected. Meanwhile, the SPI-4 pattern was not soluble in the good solvents for the pristine SPI-4 resin, such as NMP and DMAc. All these experimental results further confirm the occurrence of the photo-crosslinking reactions during the photolithographic processing.

## 4. Conclusions

A new methodology for forming fine patterns on metal substrates via the standard photolithography procedure using the solvent-free photosensitive PI NFMs as the starting materials was proposed and performed. Clear negative patterns were successfully fabricated on aluminum substrates from the electrospun auto-sensitive PSPI NFMs derived from BTDA, TMMDA, and the TSN monomers. The incorporation of TSN components into the pristine PI-1 (BTDA-TMMDA) increased the *T*_g_ and high-temperature dimensional stability of the copolymers. The electrospun PI NMFs showed good sensitivity to the emitting wavelength of high-pressure mercury lamps. The porous SPI-4 NFM patterns showed a thickness of around 15 μm and fine profiles at the line width of around 100 μm. Good comprehensive properties of the developed PI NFM patterns might be good candidates for advanced absorption, filtration, wearable display, high-frequency telecommunication, and other high-tech applications.

## Figures and Tables

**Table 1:** Formula for the PI and SPI synthesis.

PI	BTDA (g, mol)	TMMDA (g, mol)	TSN (g, mol)	NMP (g)	Ac_2_O (g, mol)	Pyridine (g, mol)
PI-1	16.1115, 0.05	12.7185, 0.05	0	69.2	25.5, 0.25	15.8, 0.20
SPI-2	16.1115, 0.05	12.0826, 0.0475	0.6859, 0.0025	86.6	25.5, 0.25	15.8, 0.20
SPI-3	16.1115, 0.05	11.4467, 0.0450	1.3717, 0.0050	86.8	25.5, 0.25	15.8, 0.20
SPI-4	16.1115, 0.05	10.8107, 0.0425	2.0576, 0.0075	86.9	25.5, 0.25	15.8, 0.20
SPI-5	16.1115, 0.05	10.1748, 0.0400	2.7434, 0.0100	87.1	25.5, 0.25	15.8, 0.20
SPI-6	16.1115, 0.05	9.5389, 0.0375	3.4293. 0.0125	87.2	25.5, 0.25	15.8, 0.20

**Table 2:** Inherent viscosities, molecular weights, and solubility of PI resins.

	(*η*)_inh_ ^1^ (dL g^−1^)	Molecular Weight ^2^			Solubility ^3^				
		*M*_n_ (×10^4^ g mol^−1^)	*M*_w_ (×10^4^ g mol^−1^)	PDI	NMP	DMAc	GBL	CPA	THF
PI-1	1.00	5.06	8.98	1.77	++	++	−	++	++
SPI-2	0.78	4.70	8.83	1.88	++	++	−	++	++
SPI-3	0.62	3.80	6.97	1.83	++	++	−	++	++
SPI-4	0.56	3.43	6.03	1.76	++	++	−	++	+
SPI-5	0.50	3.16	5.46	1.73	++	++	−	++	+
SPI-6	0.47	2.87	5.13	1.79	++	++	−	++	+

^1^ Inherent viscosities measured with a 0.5 g dL^−1^ PI solution in NMP at 25 °C; ^2^ *M*_n_: number average molecular weight; *M*_w_: weight average molecular weight; PDI: polydispersity index, PDI = *M*_w_/*M*_n_; ^3^ ++: Soluble; +: partially soluble; −: insoluble. GBL: γ-butyrolactone; CPA: cyclopentanone; THF: tetrahydrofuran.

**Table 3:** Thermal properties of PI and SPIs.

	*T*_g_ ^1^ (°C)	*T*_5__%_ ^2^ (°C)	*T*_10__%_ ^2^ (°C)	*R*_w7__5__0_ ^3^ (%)	CTE ^4^ (×10^−6^/K)
PI-1	347.6	530.2	568.5	72.3	56.5
SPI-2	366.7	516.4	564.6	65.7	55.7
SPI-3	337.6	527.8	568.4	66.5	54.0
SPI-4	366.2	521.6	566.3	66.7	53.3
SPI-5	351.1	524.0	568.1	67.0	51.0
SPI-6	359.6	507.6	558.3	64.0	50.9

^1^ *T*_g_: Glass transition temperatures measured by TMA via PI films; ^2^*T*_5%_, *T*_10%_: Temperatures at 5% and 10% weight loss, respectively; ^3^ *R*_w750_: Residual weight ratios at 750 °C in nitrogen; ^4^ CTE: linear coefficient of thermal expansion measured with the PI films in the temperature range of 50–250 °C.

**Table 4:** Optical data of PI-1 and SPI NFMs.

	*R*_365_ ^1^ (%)	*R*_436_ ^1^ (%)	*L* ^* 2^	*a* ^* 2^	*b* ^* 2^	WI ^3^ (%)
PI-1	34.2	85.3	91.98	−1.31	6.34	89.69
SPI-2	24.4	84.0	93.23	−1.65	5.25	91.28
SPI-3	21.1	82.5	93.17	−1.65	6.26	90.59
SPI-4	8.2	68.7	89.36	−2.87	9.73	85.30
SPI-5	7.6	69.8	90.72	−3.37	11.72	84.68
SPI-6	6.1	66.4	89.72	−3.42	11.82	83.97

^1^ *R*_365_*, R*_4__36_: Optical reflectance at the wavelength of 365 and 436 nm, respectively; ^2^ L^*^, a^*^, b^*^, see Section 2.2; ^3^ WI: whiteness index.

## Data Availability

Data are contained within the article.
